# Correlations of psychological distress with plasma cytokine levels and gene mutations in acral and non-acral melanoma

**DOI:** 10.3389/fpsyt.2022.1024206

**Published:** 2022-11-03

**Authors:** Hong Euy Kim, Hyun Jung Shim, Chaeyong Jung, Il Seon Shin, Jae-Min Kim, Sook Jung Yun

**Affiliations:** ^1^Department of Dermatology, Chonnam National University Medical School, Gwangju, South Korea; ^2^Department of Hemato-Oncology, Chonnam National University Medical School, Gwangju, South Korea; ^3^Department of Anatomy, Chonnam National University Medical School, Gwangju, South Korea; ^4^Department of Psychiatry, Chonnam National University Medical School, Gwangju, South Korea

**Keywords:** melanoma, anxiety, depression, quality of life, cytokine, gene mutation

## Abstract

**Background:**

The presence of psychological distress has negatively affected the course and prognosis of melanoma. Psychological distress is influenced by cytokines and gene mutations, particularly in cancer, but no studies have investigated this phenomenon in melanoma patients. This study investigated the correlations of psychological distress, plasma cytokine levels, and gene mutations in melanoma patients, focusing on melanoma sites and TNM stages.

**Methods:**

This study prospectively evaluated melanoma patients who visited Chonnam National University Hwasun Hospital from September 2020 to March 2021. Melanoma sites were divided into acral and non-acral sites. Anxiety and depression were evaluated using the Hospital Anxiety and Depression Scale, and quality of life was evaluated with EuroQol-5 Dimensions. Plasma cytokine levels, and depression- and cytokine-related gene mutations were analyzed.

**Results:**

This study included 151 melanoma patients. Anxiety was found in 14.6% of the patients, and depression in 29.8%. The melanoma sites were not significantly associated with anxiety, depression, or quality of life. However, psychological distress was significantly associated with the plasma cytokines IL-2, IL-4, IL-5, IL-10, IL-12, TNF-α and IFN-γ. *COMT, SLC6A4, SLC6A3, and IL-12b* gene mutations were also associated with melanoma sites and TNM stage, anxiety, and QOL.

**Conclusion:**

Psychological distress was associated with plasma cytokine levels and depression- and cytokine-related gene mutations. Using psychiatric intervention and emotional support, cytokine levels related to melanoma can be changed, which may have positive effects on the prognosis and treatment of melanoma. More careful follow-up, evaluation, and management are needed for patients with gene mutations.

## Introduction

The presence of psychological distress has negative effects on the course and prognosis of melanoma (1). The reported prevalence of anxiety is 32∼40% in melanoma and that of depression is 15∼33% (2, 3), and these patients have a poor quality of life (QOL) (4). Cytokine changes are related to anxiety, depression, and QOL (5–7). These include immune-modulating cytokines such as granulocyte-macrophage colony-stimulating factor (GM-CSF), anti-inflammatory cytokines such as interleukin-4 (IL-4), IL-5, IL-10, and IL-13, and pro-inflammatory cytokines such as IL-2, IL-12, interferon-γ (IFN-γ), and tumor necrosis factor-α (TNF-α). Cytokine changes are associated with anxiety, depression, and QOL in patients with various tumors, including breast and colorectal cancers (8–11). However, few studies have investigated this association in patients with melanoma.

Individual differences in cytokine production have genetic origins, affected by the transcriptional activity of cytokine gene mutations (12). Numerous studies have focused on the genetic factors of psychological distress, especially depression (9, 13, 14). Therefore, cytokine- and depression-related genes, which can cause changes in cytokine production associated with cancer progression or treatment, may confer risks of psychological distress in cancer. To our knowledge, however, no studies have tested this hypothesis in melanoma patients.

Therefore, this study investigated the correlations of psychological distress with plasma cytokine levels, and the associations of psychological distress and cytokine- and depression-related gene mutations according to the anatomic sites and TNM stages of melanoma.

## Materials and methods

### Study population

The study enrolled 151 patients with melanoma who visited Chonnam National University Hwasun Hospital from September 2020 to March 2021. All patients were confirmed to have melanoma clinically and histopathologically. Exclusion criteria included (1) patients who do not consent to study participation; (2) Patients who were not confirmed to have melanoma clinically or histopathologically; and (3) patients who are unable to make their own decisions (e.g., newborns and infants). This study was approved by the Chonnam National University Hospital Institutional Review Board (CNUH 2020-196). All participants provided informed consent. The study was conducted in accordance with the Declaration of Helsinki.

### Demographic characteristics

Demographic and clinicopathological characteristics were obtained from the medical records and clinical photographs. Melanoma staging was based on the eighth edition of the American Joint Committee on Cancer melanoma staging system, and the TNM stage was divided into melanoma *in situ* (stage 0) and stages I to IV (15). The abbreviation ‘TNM’ refers to primary tumor (T), nodes (N), and metastases (M). ‘Nodes’ indicates whether the neighboring regional lymph nodes are affected, and ‘metastases’ describes whether distant metastases have been found. We analyzed the TNM stage at the time of the survey. The anatomic sites of melanoma were classified into acral (palm, sole, and nails) and non-acral (head and neck, trunk and extremities, and genitalia) sites.

### Evaluations of psychological symptoms and quality of life

The Hospital Anxiety and Depression Scale (HADS), an instrument used internationally for assessing anxiety and depression, was used (16). This scale comprises two seven-item subscales: the anxiety (HADS-A) and depression (HADS-D) subscales. Each item is scored on 4-point scale (0–3), resulting in a total score of 0–21 for each subscale, with higher scores indicating more severe disease. A score of > 7 suggests clinically relevant anxiety or depression. The EuroQol-5 Dimensions (EQ-5D) is a standardized tool for measuring the health-related QOL (17). It consists of five dimensions: mobility, self-care, usual activities, pain or discomfort, and anxiety or depression. Each dimension has three-point rating scale (1–3), with higher scores indicating a lower QOL. These questionnaires were handed out to patients and self-administered.

### Cytokine analyses

Plasma cytokine levels were measured using a multiplex assay on the Luminex 100™ system (Luminex, Austin, TX, USA) according to the manufacturers’ instructions. Immune-modulating (GM-CSF), anti-inflammatory (IL-4, IL-5, IL-10, and IL-13), and pro-inflammatory (IL-2, IL-12, IFN-γ, and TNF-α) cytokines were analyzed.

### Cytokine- and depression-related gene mutation analyses

Whole exome sequencing was performed on the participants’ venous blood at Macrogen (Korea), using an Illumina NovaSeq platform, according to manufacturer’s protocols. Cytokine- and depression-related gene mutations were anayzed, including solute carrier family 6 member 4 (*SLC6A4*), solute carrier family 6 member 3 (*SLC6A3*), *HTR2A* (encodes the serotonin receptor), *DRD4* (encodes the dopamine receptor), catechol-o-methyl transferase (*COMT*), tyrosine hydroxylase (*TH*), and monoamine oxidase A (*MAOA*).

### Statistical analysis

Differences in age, sex, plasma cytokine levels, psychological symptoms, and QOL were evaluated according to melanoma sites and TNM stages using *t*-tests, chi-square tests, and Mann–Whitney *U*-tests. Correlations of psychological symptoms and QOL with plasma cytokine levels were evaluated using Spearman’s rank correlation. Associations of cytokine- and depression-related gene mutations with melanoma sites and TNM stages, and psychological distress were evaluated using Mann–Whitney *U*-tests and Fisher’s exact tests. A *P*-value < 0.05 was considered significant. Statistical analyses were conducted using SPSS for Windows ver. 21.0 (SPSS, Chicago, IL, USA).

## Results

### Patient characteristics

Table 1 summarizes the patient demographics and melanoma TNM stages according to anatomic sites. Of the 151 patients, 68 (45.0%) were men and 83 (55.0%) were women. Their mean age was 64.4 ± 13.1 years. Overall, 44 (29.1%) of patients were diagnosed with melanoma *in situ*, and there were 29 (21.2%), 27 (17.9%), 28 (18.5%), 20 (13.2%) with stage I to IV, respectively. Overall, 98 (64.9%) developed acral melanomas and 53 (35.1%) developed non-acral melanomas. The patients’ baseline characteristics, including age and sex, did not differ significantly between acral and non-acral sites.

**TABLE 1:** Patient demographics, TNM stage of melanoma, plasma cytokine levels, hospital anxiety and depression scale score, and EQ-5D score by melanoma anatomic sites.

Variable	Total sample (*n* = 151)	Acral site (*n* = 98)	Non-acral site (*n* = 53)
Age, years (mean ± SD)	64.4 ± 13.1	65.3 ± 12.7	62.6 ± 13.6
**Sex, *n* (%)**			
Male	68 (45.0)	49 (50.0)	19 (35.8)
Female	83 (55.0)	49 (50.0)	34 (64.2)
**Site of lesion, *n* (%)**			
Non-acral	53 (35.1)		
Head and neck			21 (13.9)
Trunk and extremity			29 (19.2)
Genitalia			3 (2.0)
Acral	98 (64.9)		
Palm		6 (4.0)	
Sole		64 (42.4)	
Fingernail		23 (15.2)	
Toenail		5 (3.3)	
**TNM stage, *n* (%)**			
0 (melanoma *in situ*)	43 (28.4)	34 (22.5)	9 (6.0)
I	32 (21.2)	21 (13.9)	11 (7.3)
II	29 (19.2)	19 (12.6)	10 (6.6)
III	27 (17.9)	15 (9.9)	12 (7.9)
IV	20 (13.2)	9 (6.0)	11 (7.3)
**Cytokines, mean (SD); median (IQR)**			
**Immune modulating**			
GM-CSF	22.0 (15.9); 17.8 (18.7)	20.9 (15.4); 15.8 (18.1)	23.8 (16.8); 22.7 (20.1)
**Anti-inflammatory**			
IL-4	45.9 (138.1); 21.1 (27.5)	41.7 (131.9); 17.3 (23.9)*	53.1 (149.7); 26.1 (29.5)*
IL-5	5.1 (5.4); 3.8 (4.0)	4.9 (5.9); 3.5 (3.6)	5.6 (4.4); 4.8 (3.7)
IL-10	10.0 (7.3); 7.8 (7.7)	9.0 (6.0); 7.8 (6.4)	11.7 (9.0); 8.4 (12.9)
IL-13	11.0 (39.4); 4.1 (6.2)	11.0 (44.8); 3.6 (5.7)	10.9 (28.1); 4.9 (5.9)
**Pro-inflammatory**			
IL-2	3.7 (3.0); 2.8 (3.4)	3.4 (3.3); 2.2 (2.8)^†^	4.1 (2.2); 4.1 (3.7) ^†^
IL-12	4.9 (7.9); 3.7 (2.9)	3.9 (2.7); 3.3 (2.9)	6.7 (12.4); 4.1 (3.6)
IFN-γ	28.5 (29.7); 22.2 (19.0)	25.5 (17.6); 21.7 (17.5)	33.8 (43.1); 25.0 (20.7)
TNF-α	8.4 (4.1); 7.3 (3.5)	7.9 (3.4); 7.0 (3.1)	9.2 (5.0); 7.8 (5.3)
**Hospital anxiety and depression scale**			
Anxiety subscale, mean (SD); median (IQR)	4.3 (2.7); 4.0 (4.0)	4.2 (2.7); 4.0 (4.0)	4.9 (2.9); 5.0 (5.0)
No. of anxiety subscale score > 7 (%)	22 (14.6)	11 (7.3)	11 (7.3)
Depression subscale, mean (SD); median (IQR)	5.2 (3.8); 4.0 (5.0)	5.1 (3.5); 4.0 (5.0)	5.9 (3.7); 5.0 (6.0)
No. of depression subscale score > 7 (%)	45 (29.8)	28 (18.5)	17 (11.3)
EQ-5D, mean (SD); median (IQR)	6.5 (1.6); 6.0 (2.0)	6.6 (1.6); 6.0 (3.0)	6.5 (1.4); 6.0 (2.0)

SD, standard deviation; IQR, interquartile range; EQ-5D, standardized measuring tool of health-related QOL. Percentages have been rounded and may not add up to 100%. ^†^*P* < 0.01; *P < 0.05 using *t*-tests, chi-square tests, or Mann-Whitney *U* tests.

### Differences of plasma cytokine levels between acral and non-acral melanomas

Plasma cytokine levels of the patients were compared according to anatomic sites of melanoma (Table 1). As a result, IL-2 (*P* = 0.008) and IL-4 (*P* = 0.027) were statistically significantly lower in acral melanoma patients than non-acral melanoma patients, and the results showed no statistically significant difference in other cytokines.

### Anxiety, depression, and quality of life subscale

The mean anxiety subscale score was 4.3 ± 2.7, and 22 (14.6%) of patients had a score > 7 (Table 1). The mean depression subscale score was 5.2 ± 3.8, and 45 (29.8%) of patients had a score > 7. The mean EQ-5D score was 6.5 ± 1.6. The mean anxiety subscale score of acral melanoma patients was 4.2 ± 2.7, and 7.3% (*n* = 11) of patients had a score > 7. The mean depression subscale score of acral melanoma patients was 5.1 ± 3.5, and 18.5% (*n* = 28) of patients had a score > 7. The mean EQ-5D score of acral melanoma patients was 6.6 ± 1.6. The mean anxiety subscale score of non-acral melanoma patients was 4.9 ± 2.9, and 7.3% (*n* = 11) of patients had a score > 7. The mean depression subscale score of non-acral melanoma patients was 5.9 ± 3.7, and 11.3% (*n* = 17) of patients had a score > 7. The mean EQ-5D score of non-acral melanoma patients was 6.5 ± 1.4. All subscales were compared between acral site and non-acral site, and there were no significant differences.

### Correlations of plasma cytokine levels with psychological distress according to melanoma site and TNM stage

In acral melanoma, the depression was negatively correlated with the IL-2 concentration (*P* = 0.02, Rho = −0.243), but there were no significant correlations between anxiety and QOL and any of the cytokines (Table 2). In non-acral melanoma, the anxiety was negatively correlated with the IL-4 (*P* = 0.042, Rho = −0.301) and IL-12 (*P* = 0.006, Rho = −0.382) concentrations, but there were no significant correlations between depression and QOL and any of the cytokines. In TNM stage 0 or I patients, no significant correlations were found with any cytokine. However, in patients with TNM stages II to IV, the anxiety was negatively correlated with the IL-4 (*P* = 0.030, Rho = −0.265), IL-12 (*P* = 0.025, Rho = −0.267), and TNF-α (*P* = 0.014, Rho = −0.287) concentrations, and the depression (*P* = 0.046, Rho = 0.231) and low QOL (*P* = 0.025, Rho = 0.260) were positively correlated with IFN-γ concentration.

**TABLE 2:** Correlations of cytokine levels with anxiety, depression, and QOL by melanoma site and TNM stage.

		Immune								
Variables		modulating	Anti-inflammatory				Pro-inflammatory			
		GM-CSF	IL-4	IL-5	IL-10	IL-13	IL-2	IL-12	IFN-γ	TNF-α
**Anatomic site**										
**Acral (*n* = 98)**										
HADS-A	Rho	–0.153	–0.044	–0.113	–0.097	0.056	–0.193	–0.075	–0.110	–0.054
	*P*-value	0.141	0.685	0.280	0.356	0.634	0.065	0.482	0.283	0.602
HADS-D	Rho	–0.164	–0.087	–0.113	–0.025	0.075	–0.243	–0.112	–0.034	–0.071
	*P*-value	0.115	0.424	0.280	0.815	0.523	*0.020	0.289	0.737	0.492
EQ-5D	Rho	0.120	0.009	0.080	0.064	0.055	–0.008	0.161	0.035	0.119
	*P*-value	0.249	0.936	0.445	0.544	0.638	0.941	0.128	0.731	0.250
**Non-acral (*n* = 53)**										
HADS-A	Rho	–0.033	–0.301	0.050	0.040	0.001	–0.147	–0.382	0.190	–0.162
	*P*-value	0.814	*0.042	0.732	0.788	0.993	0.305	^†^0.006	0.174	0.248
HADS-D	Rho	0.151	–0.228	0.017	0.054	0.019	–0.010	–0.221	0.134	–0.128
	*P*-value	0.285	0.127	0.905	0.717	0.903	0.943	0.123	0.338	0.362
EQ-5D	Rho	–0.153	–0.044	–0.113	–0.097	0.056	–0.193	–0.075	–0.110	–0.054
	*P*-value	0.141	0.685	0.280	0.356	0.634	0.065	0.482	0.283	0.602
**TNM stage**										
**TNM stage 0–I (*n* = 75)**										
HADS-A	Rho	–0.119	0.017	0.015	–0.067	–0.014	–0.085	–0.029	–0.104	0.128
	*P*-value	0.310	0.892	0.898	0.585	0.910	0.473	0.813	0.369	0.271
HADS-D	Rho	–0.181	–0.091	–0.064	0.001	–0.091	–0.177	–0.103	–0.172	0.005
	*P*-value	0.121	0.468	0.588	0.994	0.468	0.132	0.391	0.137	0.969
EQ-5D	Rho	–0.011	–0.093	0.039	0.042	–0.073	–0.146	0.056	–0.086	–0.049
	*P*-value	0.923	0.458	0.744	0.730	0.562	0.216	0.642	0.458	0.674
**TNM stage II-IV (*n* = 76)**										
HADS-A	Rho	–0.069	–0.265	–0.128	–0.002	0.139	–0.178	–0.267	0.128	–0.287
	*P*-value	0.566	*0.030	0.291	0.986	0.313	0.143	*0.025	0.275	*0.014
HADS-D	Rho	0.083	–0.133	–0.064	0.021	0.264	–0.057	–0.131	0.231	–0.175
	*P*-value	0.492	0.282	0.600	0.865	0.052	0.644	0.281	*0.046	0.139
EQ-5D	Rho	0.213	–0.095	0.157	0.167	0.186	0.142	0.115	0.260	0.194
	*P*-value	0.074	0.445	0.193	0.168	0.174	0.244	0.342	*0.025	0.100

HADS-A, Hospital Anxiety and Depression Scale – Anxiety Subscale; HADS-D, Hospital Anxiety and Depression Scale - Depression Subscale. EQ-5D, standardized measuring tool of health-related QOL. ^†^*P* < 0.01; *P < 0.05 using Spearman’s rank correlation.

### Correlations of plasma cytokine levels with psychological distress in acral and non-acral melanomas

The correlations of anxiety, depression and QOL with cytokine levels by the acral melanoma TNM stage were not significant for any cytokine (Table 3). However, in TNM stage 0 or I non-acral melanoma, depression was negatively correlated with IL-12 (*P* = 0.018, Rho = −0.534) concentrations, and low QOL negatively with IL-12 (*P* = 0.043, Rho = −0.468) and IL-2 (*P* = 0.023, Rho = −0.519). In stage II to IV non-acral melanoma, anxiety was negatively correlated with the IL-12 concentrations (*P* = 0.018, Rho = −0.421), depression positively with IFN-γ (*P* = 0.014, Rho = 0.425), and low QOL positively with IFN-γ (*P* = 0.008, Rho = 0.457), IL-10 (*P* = 0.027, Rho = 0.397), and IL-5 (*P* = 0.044, Rho = 0.363).

**TABLE 3:** Correlations of anxiety, depression, and QOL with cytokine levels by acral and non-acral melanoma TNM stage.

		Immune								
Variables		modulating	Anti-inflammatory				Pro-inflammatory			
		GM-CSF	IL-4	IL-5	IL-10	IL-13	IL-2	IL-12	IFN-γ	TNF-α
**Acral site (*n* = 98)**										
**TNM stage 0–I (*n* = 56)**										
HADS-A	Rho	–0.120	0.067	–0.036	0.004	–0.029	–0.152	0.042	–0.148	0.181
	*P*-value	0.381	0.644	0.796	0.980	0.847	0.268	0.767	0.277	0.182
HADS-D	Rho	–0.165	–0.029	–0.037	0.120	0.017	–0.177	0.041	–0.101	0.088
	*P*-value	0.228	0.842	0.790	0.392	0.908	0.197	0.774	0.458	0.521
EQ-5D	Rho	0.080	0.029	0.093	0.109	–0.051	–0.130	0.235	–0.003	0.070
	*P*-value	0.563	0.844	0.504	0.436	0.731	0.345	0.094	0.984	0.607
**TNM stage II-IV (*n* = 42)**										
HADS-A	Rho	–0.198	–0.209	–0.216	–0.219	0.211	–0.273	–0.200	–0.024	–0.352
	*P*-value	0.228	0.214	0.187	0.180	0.282	0.102	0.222	0.882	0.026
HADS-D	Rho	–0.141	–0.184	–0.221	–0.189	0.249	–0.314	–0.263	0.077	–0.279
	*P*-value	0.391	0.277	0.177	0.250	0.201	0.058	0.106	0.629	0.081
EQ-5D	Rho	0.135	–0.043	0.044	0.056	0.212	0.106	0.112	0.131	0.183
	*P*-value	0.414	0.802	0.792	0.737	0.279	0.534	0.497	0.409	0.260
**Non-acral site (*n* = 53)**										
**TNM stage 0–I (*n* = 19)**										
HADS-A	Rho	–0.130	–0.287	0.138	–0.332	–0.072	–0.164	–0.204	0.056	–0.017
	*P*-value	0.584	0.280	0.574	0.209	0.768	0.503	0.402	0.813	0.942
HADS-D	Rho	–0.199	–0.352	–0.211	–0.395	–0.411	–0.440	–0.534	–0.317	–0.322
	*P*-value	0.401	0.181	0.387	0.130	0.080	0.059	*0.018	0.151	0.055
EQ-5D	Rho	–0.333	–0.429	–0.196	–0.250	–0.246	–0.519	–0.468	–0.333	–0.435
	*P*-value	0.151	0.097	0.421	0.350	0.309	*0.023	*0.043	0.151	0.055
**TNM stage II–IV (*n* = 34)**										
HADS-A	Rho	0.032	–0.304	0.012	0.205	0.073	–0.104	–0.421	0.311	–0.231
	*P*-value	0.864	0.102	0.948	0.269	0.718	0.570	*0.018	0.078	0.197
HADS-D	Rho	0.325	–0.069	0.169	0.267	0.238	0.176	–0.017	0.425	–0.085
	*P*-value	0.070	0.719	0.363	0.147	0.231	0.334	0.929	*0.014	0.639
EQ-5D	Rho	0.344	–0.160	0.363	0.397	0.157	0.190	0.173	0.457	0.232
	*P*-value	0.054	0.398	*0.044	*0.027	0.435	0.297	0.352	^†^0.008	0.193

HADS-A, Hospital Anxiety and Depression Scale - Anxiety Subscale; HADS-D, Hospital Anxiety and Depression Scale - Depression Subscale. EQ-5D, standardized measuring tool of health-related QOL. ^†^*P* < 0.01; *P < 0.05 using Spearman’s rank correlation.

### Associations of cytokine- and depression-related gene mutations with anatomic sites, TNM stages, and psychological distress

There were no significant associations of cytokine-related gene mutations with melanoma sites and TNM stages (Table 4). However, the *COMT* gene mutation showed a significant association in acral melanoma (*P* = 0.016), and the *SLC6A4* gene mutation had a significant association in patients with TNM stage 0 or I (*P* = 0.008). There were no significant associations of any gene mutations with depression (Table 5). However, the *IL-12b* gene mutation was significantly associated with anxiety (*P* = 0.031), and the *SLC6A3* gene mutation was significantly associated with low QOL (*P* = 0.017).

**TABLE 4:** Associations of cytokine- and depression-related gene mutations with anatomic sites and TNM stage of melanoma.

Gene mutations	Anatomic site					TNM stage				
	Acral (*n* = 98)		Non-acral (*n* = 53)		*P-value*	TNM stage 0–I (*n* = 75)		TNM stage II–IV (*n* = 76)		*P-value*
	Present	Absent	Present	Absent		Present	Absent	Present	Absent	
**Cytokine-related genes**										
*IL-4*	5	51	4	94	0.166	4	71	5	71	0.492
*IL-5*	2	52	1	97	0.282	2	73	1	75	0.505
*IL-10*	1	53	1	97	0.580	0	75	2	74	0.245
*IL-12a*	0	47	2	96	0.420	0	75	2	74	0.245
*IL-12b*	6	41	7	91	0.279	7	68	6	70	0.510
**Depression-related genes**										
*SLC6A4*	26	72	12	34	0.375	25	50	13	63	^†^0.008
*SLC6A3*	38	60	19	51	0.431	30	45	27	49	0.272
*HTR2A*	5	93	2	34	0.529	2	73	5	71	0.216
*DRD4*	37	61	19	44	0.480	33	42	23	53	0.073
*COMT*	34	64	9	44	*0.016	18	57	25	51	0.128
*TH*	11	87	3	50	0.206	8	67	6	70	0.400
*MAOA*	13	85	11	42	0.166	14	61	10	66	0.264

^†^*P* < 0.01; *P < 0.05 by using Fisher’s exact tests.

**TABLE 5:** Associations of cytokine- and depression-related gene mutations with anxiety, depression, and QOL.

Gene mutations	HADS-A score (mean ± SD)			HADS-D score (mean ± SD)			EQ-5D score (mean ± SD)		
	Present	Absent	*P-value*	Present	Absent	*P-value*	Present	Absent	*P-value*
**Cytokine-related genes**									
*IL-4*	4.7 ± 2.6	4.4 ± 2.8	0.755	6.0 ± 4.4	5.3 ± 3.5	0.695	6.4 ± 2.0	6.6 ± 1.5	0.444
*IL-5*	7.3 ± 4.4	4.4 ± 2.7	0.073	7.6 ± 1.5	5.3 ± 3.6	0.184	6.3 ± 0.6	6.5 ± 1.6	0.902
*IL-10*	4.5 ± 6.4	4.4 ± 2.7	0.967	5.5 ± 7.8	5.4 ± 3.6	0.915	7.5 ± 3.5	6.5 ± 1.5	0.805
*IL-12a*	3.0 ± 1.4	4.5 ± 2.8	0.459	5.5 ± 2.1	5.4 ± 3.6	0.805	6.5 ± 2.1	6.6 ± 1.6	0.941
*IL-12b*	2.9 ± 2.7	4.6 ± 2.7	*0.031	3.9 ± 2.8	5.5 ± 3.6	0.086	6.9 ± 1.7	6.5 ± 1.5	0.382
**Depression-related genes**									
*SLC6A4*	5.1 ± 2.7	4.2 ± 2.7	0.091	6.2 ± 3.7	5.1 ± 3.5	0.081	7.0 ± 1.6	6.4 ± 1.6	0.083
*SLC6A3*	4.3 ± 2.6	4.5 ± 2.9	0.721	6.1 ± 4.0	5.0 ± 3.3	0.110	7.0 ± 1.7	6.3 ± 1.5	*0.017
*HTR2A*	4.0 ± 2.1	4.5 ± 2.8	0.834	6.7 ± 2.9	5.3 ± 3.6	0.208	6.3 ± 1.1	6.6 ± 1.6	0.841
*DRD4*	3.9 ± 2.7	4.8 ± 2.7	0.051	4.7 ± 3.1	5.8 ± 3.8	0.092	6.3 ± 1.3	6.7 ± 1.7	0.230
*COMT*	4.3 ± 3.0	4.5 ± 2.6	0.653	5.3 ± 3.5	5.4 ± 3.6	0.805	6.6 ± 1.7	6.6 ± 1.5	0.630
*TH*	4.6 ± 2.6	4.4 ± 2.8	0.718	5.7 ± 4.0	5.3 ± 3.6	0.784	7.0 ± 1.9	6.5 ± 1.5	0.364
*MAOA*	4.5 ± 4.4	4.4 ± 2.7	0.912	5.8 ± 3.8	5.3 ± 3.6	0.559	6.3 ± 1.3	6.6 ± 1.6	0.473

HADS-A, Hospital Anxiety and Depression Scale-Anxiety Subscale; HADS-D, Hospital Anxiety and Depression Scale-Depression Subscale. EQ-5D, standardized measuring tool of health-related QOL. *P < 0.05 using Mann–Whitney-*U* test.

## Discussion

A few recent studies have examined prevalence of anxiety and depression in melanoma patients. Tas et al. (2) reported that about one third of Turkish melanoma patients had anxiety and depression, and Beesley et al. (3) reported anxiety in 32% of Australian melanoma patients and depression in 15%, and their emotional QOL was poor compared to the general population. In our study, 14.9% of melanoma patients had clinically relevant anxiety, and 29.8% had depression, similar to slightly lower than previous results (2, 3). No studies have reported associations of psychological distress with anatomic sites of melanoma. Because acral melanoma is the most common subtype in Asian populations (18), we enrolled more patients with acral melanomas. However, the melanoma anatomic sites were not associated with anxiety, depression or QOL. Therefore, we postulate that melanoma itself, not its anatomic sites, causes psychological distress in melanoma patients.

Several studies have examined cytokines in melanoma, including cytokine expression and application to treatments (19, 20). To our knowledge, this is the first investigation to find associations of melanoma anatomic sites and TNM stages with psychological distress by comparing plasma cytokine levels. We found that IL-4 and IL-12 were significantly associated with anxiety. According to Kawakami et al. (21) IL-4 promotes the growth of tumor-infiltrating lymphocytes, which are cytotoxic for human autologous melanoma, and can be used in cellular melanoma immunotherapy. In our study, IL-4 was negatively correlated with anxiety in patients with non-acral melanoma, TNM stages II to IV, and non-acral melanoma with TNM stages II to IV. Our result also supports the finding that IL-4 knock-out mice display anxiety-like behavior, in comparison to wild-type mice (22). In addition, we compared plasma cytokine levels according to anatomic sites, and patients with acral melanoma showed lower levels of IL-4 than patients with non-acral melanoma. It suggests that immunotherapy using IL-4 or immune-checkpoint inhibitors may have lower therapeutic effects on the acral melanoma than non-acral melanoma, however, further research is needed on this issue.

IL-12 is a pro-inflammatory cytokine that stimulates both innate and adaptive immunity (23). In our study, IL-12 was negatively correlated with anxiety in patients with non-acral melanoma and TNM stages II to IV. IL-12 was also negatively correlated with depression in non-acral melanoma with TNM stages 0 and I, and with low QOL in non-acral melanoma TNM stages 0 and I. In addition, the *IL-12b* gene mutation was significantly associated with a low HADS-A score. Daud et al. (24) reported that plasmid IL-12 electroporation induced disease stabilization and a partial response in patients with metastatic melanoma, along with tumor necrosis and lymphocytic infiltration. Fang et al. (25) reported a significant correlation between the *IL-12b* gene mutation and serum IL-12 levels in melanoma patients, and the *IL-12p40* mutation contributed to melanoma susceptibility and influenced patient outcome.

Due to its antitumor activity, a high-dose bolus of recombinant IL-2 has been used to treat melanoma for decades (26). IL-2, a T-cell growth factor, was negatively correlated with depression in patients with acral melanoma, and with a low QOL in non-acral melanoma with TNM stages 0 and I, in our study. Therefore, the prognosis of melanoma might be improved by increasing IL-2 levels by relieving depression and increasing QOL through psychological support. Also, patients with acral melanoma showed lower levels of IL-2 than non-acral melanoma. It suggests that immunotherapy using IL-2 may have different effects depending on the anatomical site of melanoma, and further studies are needed on this issue.

In our study, TNF-α was negatively correlated with anxiety at TNM stages II to IV. However, IFN-γ was positively correlated with depression at TNM stages II to IV patients overall and in non-acral melanoma, and with a low QOL in TNM stage II to IV patients. TNF-α has potent antitumor activity via endothelial cell activation and vascular damage, resulting in hemorrhagic necrosis (27). The number of TNF-α receptors on malignant cells increases when cultured with IFN-γ (28). Lienard et al. (27) demonstrated that IFN-γ therapy in combination with TNF-α produced good responses in melanoma patients. Thus, the prognosis of melanoma might be improved by increasing the TNF-α levels through reducing anxiety with psychiatric intervention, along with surgical and systemic treatments. Depression and IFN-γ were positively correlated in our study, which is consistent with reports that plasma IFN-γ level increased in patients with depression (5).

IL-5 and IL-10 were positively associated with a low QOL in non-acral melanoma TNM stage II to IV. Although the relationship between melanoma and IL-5 has not been studied, IL-5 facilitates lung metastasis by modulating the immune environment (29). IL-10 also inhibits the production of many cytokines and suppresses tumor growth and metastasis of human melanoma cells by inhibiting macrophage-derived angiogenic factors (30). Based on these findings, the prognosis of melanoma may be improved by reducing the IL-5 levels through improving QOL via sufficient physical and emotional support. Further research on the relationship between the melanoma prognosis and IL-10 according to the QOL is needed.

We also assessed depression-related gene mutations. Several studies have reported that the *COMT* mutation is associated with the response to anxiety, depression (31), and Alzheimer’s disease (32). Studies have examined the association between melanoma pigmentation and *COMT* activity (33, 34). In our study, the *COMT* gene mutation was significant associated in patients with acral melanoma, which usually appear as markedly pigmented lesions, but the association with the *COMT* gene mutation needs further study.

The *SLC6A4* gene mutation showed a significant association only in patients with TNM stage 0 or I; it was not associated with psychological distress. This gene encodes serotonin transporter protein, which is the target of many antidepressants (35), and several studies have reported an association between the *SLC6A4* gene mutation and psychiatric disorders (36, 37). Other studies have linked the *SLC6A3* gene mutation with psychiatric disorders such as depression, bipolar disorder, and alcoholism (38). In our study, a low QOL was associated with the *SLC6A3* mutation. No studies have reported on the relationship between the *SLC6A3* gene mutation and melanoma or QOL, and further research in this area is needed.

This study had several limitations. First, due to low prevalence of melanoma, we had no choice but to include almost all melanoma patients who visited our hospital as study subjects. They were included in the study regardless of whether they were first visits to the hospital, before or after surgery, and whether they received chemotherapy, immune checkpoint inhibitor therapy, or radiation therapy, which may had affected the plasma cytokine levels, so we had difficulty equalizing the conditions of study subjects. Second, the number of acral melanoma patients was almost twice that of non-acral melanoma patients, which may have affected the statistical analysis. Third, patients were not assessed for previous psychological disorders. Fourth, we administered an assessment scale (HADS) rather than a formal diagnostic instrument for evaluating anxiety and depression. Therefore, comorbidity of anxiety and depression could not be considered despite of their common simultaneous existence (39). In addition, we used Spearman’s rank correlation to evaluate correlations of psychological symptoms and QOL with plasma cytokine levels. However, Spearman’s rank correlation results can only show the relationship between the two, not the sequence and causality. The data may only suggest correlation, and may not demonstrate causation. Cytokines may be a bystander that are unrelated to melanoma biology and outcome, or the downstream effect of melanoma biology rather than a contributor to melanoma biology.

In conclusion, we found that psychological distress was associated with the cytokines IL-2, IL-4, IL-5, IL-10, IL-12, TNF-α, and IFN-γ. *COMT, SLC6A4, SLC6A3*, and *IL-12b* gene mutations were also associated with melanoma sites and TNM stage, anxiety, and QOL. Since this is the first report on supporting the inflammatory hypotheses of psychological symptoms in patients with melanoma, further replication and longitudinal studies are needed to confirm the findings. Moreover, future interventional studies would be helpful to investigate whether psychiatric treatment with emotional support can change cytokine levels related to melanoma, which may have positive effects on the prognosis and treatment of melanoma. More careful follow-up, evaluation, and management are indicated for those with gene mutations.

## Data availability statement

The data presented in the study are deposited in the National Library of Medicine repository, at the link: https://dataview.ncbi.nlm.nih.gov/object/ with the accession numbers SAMN31327467, SAMN31327468, SAMN31327469, SAMN31327470 etc. up to SAMN31327617.

## Ethics statement

The studies involving human participants were reviewed and approved by the Chonnam National University Hospital Institutional Review Board (CNUH 2020-196). The patients/participants provided their written informed consent to participate in this study.

## Author contributions

HK and SY: conceptualization and writing—original draft preparation. HK, HS, J-MK, and SY: formal analysis. J-MK: funding acquisition. HK, CJ, IS, J-MK, and SY: investigation. J-MK and SY: methodology and writing—review and editing. HS, CJ, IS, J-MK, and SY: validation. All authors contributed to the article and approved the submitted version.
